# Early Prediction of Cardio Vascular Disease (CVD) from Diabetic Retinopathy using improvised deep Belief Network (I-DBN) with Optimum feature selection technique

**DOI:** 10.1186/s12872-024-04374-0

**Published:** 2025-01-18

**Authors:** T. K. Revathi, B. Sathiyabhama, S Kaliraj, Vidhushavarshini Sureshkumar

**Affiliations:** 1 Department of CSE, Sona College of Technology, Salem, Tamilnadu India; 2 Department of Information and Communication Technology, Manipal Institute of Technology, Manipal Academy of Higher Education, Manipal, 576104 Karnataka India; 3 Department of Computer Science and Engineering, SRM Institute of Science and Technology, Vadapalani Campus, Chennai, India

**Keywords:** Cardiovascular disease, Diabetic retinopathy, Deep belief network, Prediction

## Abstract

Cardio Vascular Disease (CVD) is one of the leading causes of mortality and it is estimated that 1 in 4 deaths happens due to it. The disease prevalence rate becomes higher since there is an inadequate system/model for predicting CVD at an earliest. Diabetic Retinopathy (DR) is a kind of eye disease was associated with increasing risk factors for all-causes of CVD events. The early diagnosis of DR plays a significant role in preventing CVD. However, there are many works have been carried out on classification of the disease but they focused less on feature selection and increasing the accuracy of the model. The proposed work introduces Improvised Deep Belief Network named I-DBN to resolve the above mentioned problems and mainly to concentrate on improving the entire performance of the model leading to the unbiased output. We used Principal Component Analysis (PCA) and Particle Swarm Optimization (PSO) algorithm for feature extraction and selection respectively. Five performance metrics have been used to assess the proposed model. The results of I-DBN outperform other state-of-the-art methods. The result validation ensures that I-DBN can deliver trustworthy recommendations to doctors to treat the patients by enhancing the accuracy of CVD prediction up to 98.95%.

## Introduction

In India, mortality rate is increases sue to non-communicable diseases. Report from World Health Organization (WHO) says that, CVD is one of the primary causes of death and disabilities in most cases in the world among human nowadays [1]. It is not a single disease rather a collection of diseases such as CVD, angina, hypertension, stroke and DR which affects the vascular system, cardiac and sensory systems [2]. The primary risk factors of CVD are history of family in terms of disease, demographic characteristics, smoking, physical inactivity, obesity, hypertension and diabetes [3]. DR is an eye disease that affects the retina caused due to diabetes mellitus [4]. There are two types of diabetes mellitus, one is Type1 Diabetes (T1D) caused due to insufficient level of insulin in the blood cell. Another is Type2 Diabetes (T2D) categorized by sugar level in the blood. Sometimes, Diabetes mellitus lead to vision loss to the eye. The patients having these types of complications become blind if they left unprocessed. The statistical report in 2017, there are 425 million grown-ups affected with diabetes and the affected people count is gradually increases to 629 million by 2045 [5]. People affected with the DR are most likely to have CVD. CVD can be predicted by the presence of hemorrhages, micro aneurysms, and exudates and morphological changes in the eye. DR plays a significant part in the prediction and stratification of CVD. DR is identified by the weakening of blood vessels and exudates. Exudates are fluids composed of serum, pus discharge out from the infected areas/dead cells. It can also be found by the presence of micro aneurysms. Micro aneurysms are tiny bulges happens in the blood vessels of retina [6, 7].

Most people belonging to the age group 40 to 59 are affected with T2D. Around 212 million people were affected with this risk category, wherein 50% of the people are completely unaware of their symptoms that cause the disease [5]. Hence, DR has a higher chance to become a major health hazard all over the world. To avoid this it has to be predicted at an early stage. The primary factors like Obesity, irregular diet, and less body activity are responsible for T2D. However, it is significant to know that DR occurs to the patients those who have only diabetes for about ten years. The reason behind the cause of DR is the patients are remains unaware, ignores treating the disease and lazy in examining eye properly. Regular health check-ups and treatment of diabetes could helps in predicting the Diabetic Retinopathy earlier and prevents from CVD [8, 9].

In diabetics mellitus, usually our humans body transforms the blood glucose levels into an energy which activates the regular body functioning. Variations in sugar levels cause accumulation of excess glucose in the blood vessels. Due to this the blood flow gets affected to the various parts of the body, particularly eye. This leads to hyperglycemia.

T1 D blood sugar/glucose level to be controlled in human body by identifying the hormone called insulin. The patient affected with T1D would require taking up insulin injection regularly since body loses the capacity to generate enough insulin to the body. They become dependent on insulin injection in their life otherwise it leads to irregular sugar levels in the bloods which may cause multiple health problems [10, 11].

T2 D patient affected with T1D is not insulin dependent. In this case, there is no problem in producing enough insulin in the body. But it fails in utilizing insulin for energy level conversion. Since there is no enough energy produced, the body generates abnormal and excess blood sugar level with unutilized energy [10].

In both T1 D and T2 D, the irregular or abnormal generation of sugar level suspects a patient remains Diabetic Retinopathy. Excess amount of glucose block the blood vessels of retina hence the patient feel problem in their eye vision. Due to this, the patients may get symptoms of DR includes cataract, vision fluctuation, floating eye, eye spots, blurred and double vision. Angiography treatment is conducted to diagnose the disease. During the angiography the patient injected with a dye in his/her arm. The dye passes into the blood vessels of retina for detecting the blood leakages in the vessel. Also it finds the diabetic blood vessel changes. Higher sugar level spoils the vessel which leads to bleeding. At the early stage of DR, inflammation happened to the retinal vessel and excess blood gets accumulated in it. At an advanced stage, leakages in the blood vessels cause serious eye problems even leads to blind vision [12]. The working nature of the modern ML model as DBN had much contribution towards mammogram screening and other disease predictions thus reduces the trouble data interpretation [13]. DBN has the capability to train the model with lesser set of labeled data and also it takes comparatively short time to train the model on GPU machines [14, 15]. Over other network models DBN has a notable advantage of providing solution to gradient problems [16]. These benefits of DBN motivates the present study to contribute on,

1. Prior diagnosis of CVD using the morphological changes in eye providing a greater chance for doctors to treat and recommend medication to cure the same.
2. Gives more attention on using the most important needy feature factors by removing the irrelevant ones.
3. Focus on achieving high accuracy in classifying the risk factors of the event after the dimensionality reduction.

A Deep Belief Network is applied in this study in association with PCA and PSO for predicting prevalence of CVD by doing the classification task on UCI DR Dataset. The data gathered from UCI contains irrelevant attributes which raise burden to the model. Hence PCA is applied for extracting significant features from the dataset. To enhance the classification performance further, PSO is executed for feature reduction. There by I-DBN model will receive the most relevant features for generating improvised classification. I-DBN model’s performance is evaluated on the common metrics accuracy, precision, recall, specificity and sensitivity.

 The sections mentioned below are structured as follows: Sect. 2 represents related work, Sect. 3 the proposed methodology is introduced, Sect. 4 describes the experimental setup, Sect. 5 indicates the result and discussions and Sect. 6 concludes with scope of future work.

## Related work

The deep learning approaches are used for giving better accuracy compared to conventional model in predicting diabetic retinopathy [17]. The authors performed analysis on less volume of higher resolution images. The experimental results underlined and compare the performance ability of various Deep Learning (DL) models to predict the disease. Also the results were highlighted the models performance levels in terms of cost as well. However the author focused on analyzing the fundus image for DR only to grade macular edema.

In [18] decision made for quantifying the errors in Diabetic Retinopathy (DR) to grade the disease using DL approach. The performance kappa score metric was obtained and the developed model is compared with the other conventional model based on accuracy, sensitivity and Area under the Curve (AUC). But the authors have not focused on classification of DR in terms of finding the risk that is going to cause the cardiac disease.

In [19], data oriented DL model has been used for diagnosing DR disease. However, the model proposed needs to be fine tuned for the pre-trained CNNs on the dataset considered by them. This works focused on DR1 for fine-tuning the model to estimate the average image against training images from DR dataset.

Retina fundus colored image has been used for classifying the diseased ones from the normal healthy images [20]. They proposed Variational Mode Decomposition (VMD) for separating higher frequency elements of the image data which they considered. And also the authors coined retinal images for predicting only hemorrhages. They trained the model with textual descriptors from Discrete Wavelet Transform (DWT). This paper not focused on training the model with lesser iterations to find the appropriate features to be needed.

A kind of DL model is designed to diagnose Referable Diabetic Retinopathy (RDR) [21] and also the authors were predicted mascular edema from retinal fundus image. The authors were used the retinal images as input for detecting modest and worsen case of DR. They evaluated their model only in terms of high specificity and sensitivity. However, the authors have aimed on feature engineering. Authors were designed an algorithm to find the proper lesions of any stage of DR.

A fully Convolutional Neural Network (CNN) is implemented to train the model for crowd detection [22]. They coined DL for predicting huge variety of places, other kinds of environments and various lighting conditions. But however this work nothing addressed on health care related research project.

Heart Rate Variability (HRV) dataset were proposed for classifying DR disease using DL model [23]. The authors combined CNN with Long-Short-Term Memory (LSTM) for extracting the dynamic features of HRV data. They attained the experimental results with better accuracy in classifying HRV data. The work aimed for making the clinicians to predict the disease but they coined the conventional diagnosing tool called ECG.

A combination of CNN-LSTM [24] proposed for detecting diabetes mellitus automatically. The authors diagnosed the diabetes disease by analyzing Heart Rate Variability (HRV) from ECG signals. The authors were discussed about the TypeI Diabetes and Type II diabetes data for predicting the event. The experiments were conducted with the ECG signals gathered from 20 normal and 20 diabetes patients in a supine position. However the authors were used Diabetes dataset for only for predicting the diabetes. In a similar research [25] conducted a study on future prognosis of diabetes using the conventional ECG signal as a diagnostic tool. In this study they were analyzed HRV and also proposed distributed DL algorithm for discriminating normal case from the person who affected with the diabetes disease.

A DL model [26] created for predicting the risk factors that causing cardiovascular disease. The authors were experimented their study with UK Biobank and EyePACS dataset. They conducted their research for estimating the changes in anatomical features of fundus image to generate each and every CVD prediction. The author conferred that in order to achieve more accurate DL model it must trained with a huge volume of dataset.

The latest DL technologies [27] proposed for grading the DR disease. To automate their system the authors were used Retinal images as input. The author mentioned that DR dataset is useful in predicting the minor changes happens in the anatomical structure of the retina. However, the author limits the research in finding the various stages of DR not on CVD.

The prediction of future DR dealt by using color fundus photographs (CFPs) [28]. They were found the various stages of DR against its severity score. Deep Convolutional Neural Networks (DCNNs) has been used for assessing the images to produce the output [29]. However this works helped in knowing the types of DR for that causes the CVD prevalence.

In [30] DBN has been used for diagnosing heart disease. Ruzzo-Tompa approach was applied for removing the unwanted features for the prediction. They evaluated their model with all the six basic performance metrics. The author handled the overfitting issue by applying the optimal feature selection methodology. They also analyzed the trained DBN model for finding different network layer’s depth.

In [31] the study explores the use of machine learning to predict the progression of diabetic retinopathy, leveraging risk factors that overlap with those affecting cardiovascular health. The work supports the approach of using DR as a predictor for systemic conditions such as CVD by identifying shared biomarkers.

In [32] the research applies convolutional neural networks to analyze retinal images, predicting DR progression and examining links to cardiovascular risk. It highlights how retinal microvascular changes, evident in DR, can be predictors for CVD, validating their relevance in early detection strategies.

In [33] the paper discusses retinal biomarkers as indicators for systemic health issues, including cardiovascular risks. Machine learning models are used to identify these biomarkers in DR patients, reinforcing the value of retinal imaging in predicting broader health outcomes like CVD.

In [34] the authors are analyzed data from diabetic retinopathy patients, this study develops machine learning algorithms for cardiovascular risk assessment, supporting the idea that DR indicators can be predictive of CVD. It shows that DR data enhances risk stratification in diabetic patients for cardiovascular complications.

In [35] the study leverages artificial intelligence for early diabetes and diabetic retinopathy detection. By applying AI models to DR screening, the work identifies key biomarkers linked to systemic health conditions, laying groundwork for broader disease prediction, including cardiovascular risks.

The authors [36] are explored AI’s role in early cancer detection, this paper highlights advanced algorithms for predictive modeling. Though focused on oncology, the findings reveal cross-disease applications in early diagnosis, which are relevant for cardiovascular and metabolic conditions.

The research paper [37] examines DR as a predictive biomarker for cardiovascular events in type 2 diabetes patients. It underscores the link between retinal health and systemic vascular risks, providing evidence that DR data can effectively signal elevated CVD risk.

The authors [38] use retinal imaging to assess cardiovascular health via AI, noting that retinal microvascular changes correlate with CVD risk. Their findings support retinal data’s predictive value in systemic health assessment and cardiovascular disease monitoring.

It is observed from the literature review that maximum of the research work focused only in predicting CVD after it happens. The limitations are as follows, various optimization algorithms were proposed for feature selection but failed to address the over fitting and under fitting problems, some optimization techniques are consuming much time to calculate the fitness function, conventional diagnosing tool ECG were used by most of the researchers and network optimization issues. To overcome these issues, this paper proposed I-DBN to prevent CVD before it occurs. Hence, we used DR dataset which is relatively proven that plays a vital role in diagnosing CVD early it happens. In addition, this work addresses the over fitting problem by training the model with optimum feature selection and then it is extended to provide reliable output for CVD prediction. I-DBN resolves the optimization problem by randomly selecting the number of suitable layers and hyper-parameter while designing the network model.

The exclusive contribution of the I-DBN model includes:

1. Optimum based feature selection algorithm is implemented with reasonable number of random state iterations for selecting the most important attributes to train the model proposed in this study.
2. The adoption of I-DBN is comparatively minimizes the training time of the model since it has the capacity to train the model with lesser set of labeled data.

## Proposed I-DBN approach

**Fig. 1:**
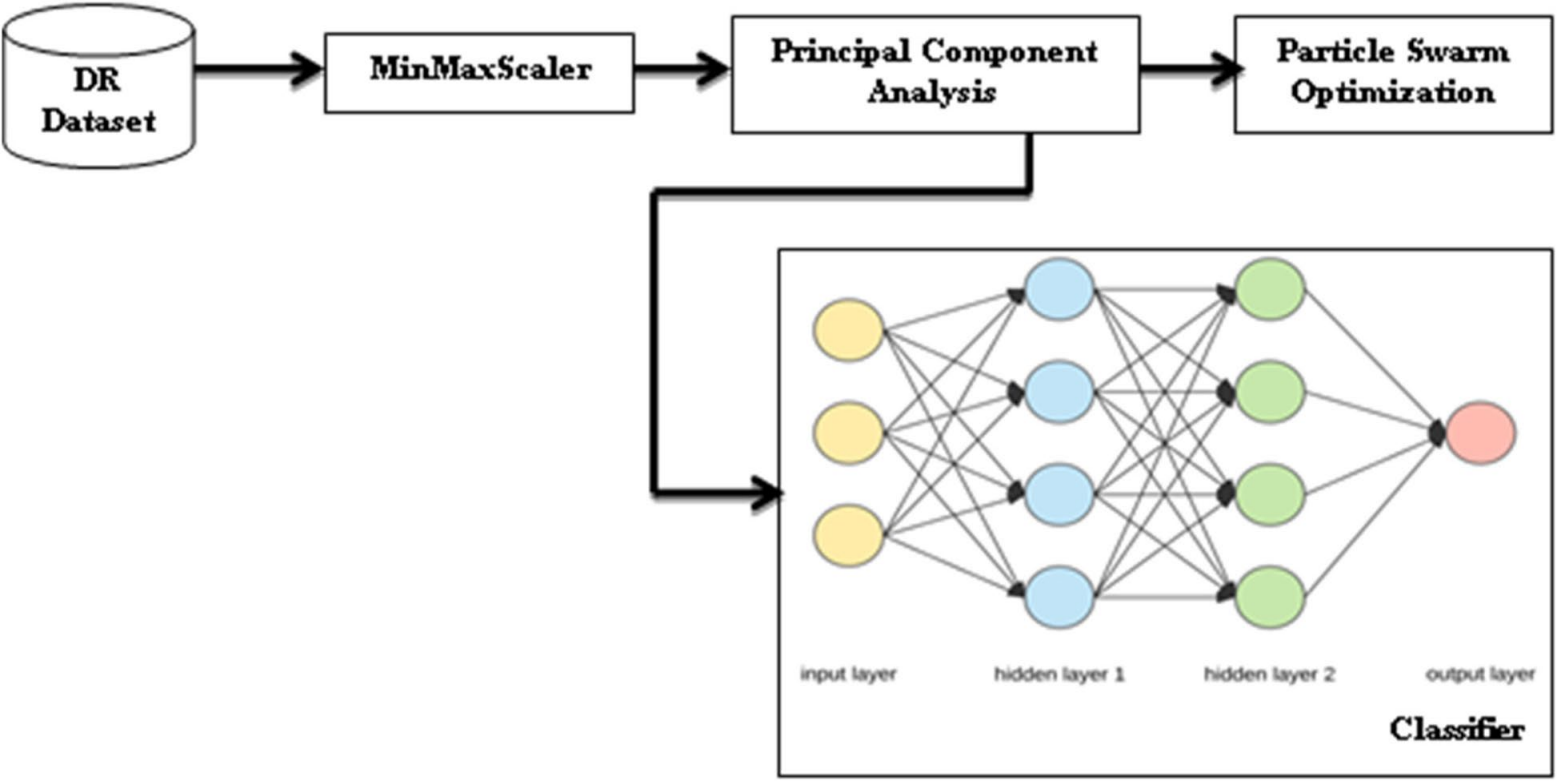
The Proposed I-DBN Model

In this work, first the standardization is done on the dataset using MinMaxScaler method. Standardization includes removing of outliers, data transformation and data normalization. Not all the features available in the dataset that affect the class label. For feature reduction PCA technique has been used [39, 40]. This dimensionality reduction technique is one of the best approaches to remove the useless features from the dataset. Then the optimized parameters were chosen by using PSO. This has been given as input to train our I-DBN model. DBN is used to classify the DR Dataset. In the last layer of the classifier, the sigmoid function has been applied as an activation function. To train and test the model 80 − 20 ratio is applied. For all epochs a batch of 80 records were provided as an input to the model. Among these 80 records, 80% were used to train the model and 20% were used to test the model. The Fig. 1 depicts the proposed I-DBN model’s work flow.

The dataset considered in this work is gathered from UCI online DR repositories. It has 1151 instances with 20 attributes. The dataset features are extracted from the Messidor dataset. The description of the attributes is mentioned in Table 1.

**Table 1:** Dataset description

Attribute no.	Description
0	The binary outcome of feature evaluation. 0 - denotes poor quality 1 – denotes required quality
1	The binary outcome of pre-screening: 1 –High risk of retinal defects 0 - indicates lack of retinal defects
2–7	Number of Microaneurysm (MA) features
8–15	It is same as attributes from 2–7 indicate number of exudates features
17	It represents Euclidean distance which is standardized to the diameter of Region of Interest
18	It represents the Optic Disc’s diameter
19	AM/FM based classification which results in binary.
20	Class_label 1 - indicates presence of DR disease Class_label 0 - indicates absence of disease

Standardization is done on the DR dataset to remove the outliers. These normalized data is involved to feature reduction by using PCA algorithm. Then the work selects the suitable features from it by using PSO algorithm. Next the relevant features are given as input to the I-DBN model to classify the DR dataset. Based on this classification report the proposed model recommends the doctor to identify the risk level seems to be cause the CVD. This work also helps the medical practitioners to treat the patients with recommended medication to protect them from CVD prevalence.

The proposed model is summarized as follows:

**Table:** 

Summary of proposed I-DBN model
***Input***: DR Dataset ***Output***: Binary Class Label 1) ***Data Standardization***: Standardization is done using MinMaxScaler Method 2) ***Feature Reduction***: PCA algorithm is used for dimensionality Reduction 3) ***Optimum Feature Selection***: Optimal parameters were chosen with PSO algorithm. These features were given as input to the DBN classifier.
***The following steps involved in optimal features selection***:
***Step 3.1): Do Initialization***
(a) Set constants$\:{x}_{\text{max},}$, s_1_, s_2_ (b) Do initialization of particle positions randomly,$\:{pp}_{o}^{i}$€ d in IR^n^ for i = 1,….,p (c) Do initialization of particle velocities randomly, 0≤$\:{v}_{0}^{i}$≤$\:{v}_{0}^{max}$for i = 1,….,p (d) Set x = 1
***Step 3.2): Optimization***
(a) By using design space coordinates$\:{pp}_{x}^{i}$, evaluate the function value$\:{f}_{k}^{i}$ (b) Check IF$\:{f}_{k}^{i}$≤$\:{f}_{best}^{i}$, THEN$\:{f}_{best}^{i}$=$\:{f}_{k}^{i}$,$\:{P}_{x}^{i}$=$\:{pp}_{x}^{i}$ (c) Check IF$\:{f}_{k}^{i}$≤$\:{f}_{best}^{k}$, THEN$\:{f}_{best}^{k}$=$\:{f}_{k}^{i}$,$\:{P}_{x}^{k}$=$\:{pp}_{x}^{i}$ (d) IF satisfied THEN, GOTO *Step3* (e) Do update of all particle velocities,$\:{v}_{x}^{i}$for i = 1,…,p (f) Do update of all particle positions,$\:{pp}_{x}^{i}$for i = 1,…,p (g) Do incrementing the value of x by 1 (h) GOTO *Step2*(a)
***Step3.3: Terminate the algorithm***
4) Classification: DBN classifier is used to classify DR.
5) Performance Evaluation: I-DBN model is evaluated by using various performance metrics. The DBN Model (with hidden layers) is shown Fig. 1
6) Performance comparison of this model with conventional ML models

### Principal component analysis

The normalized DR dataset is given as input to the PCA to reduce the features. It is a dimensionality reduction technique that describes the variance of the data in a lower dimensional representation with minimum reconstruction error [41]. PCA is a well-liked transform method and apparently the result of transformation is in direct to a feature element of original data sample. However, the PCA has the latent to find Principal Components (PCs) in selecting a number of useful features from the entire feature components. It has been achieved from the view of numerical computation by performing the PCA transformation as a numerical analysis problem [42].

It is one of the finest data mining approaches to extract the PCs in the track of data portraying larger variability. The major key aspect is noise-free and extracts the suitable patterns in the data [43]. PCA is a quantitative method for achieving feature selection. It usually creates a novel set of identifiers, called PC. Each component is a linear group of the original variables. There is no redundant information because the entire component is of orthogonal to each other. It forms an orthogonal base to the space of the data [41]. Thus we use the PCA algorithm for identifying critical novel features by using eigenvectors.

**Fig. 2:**
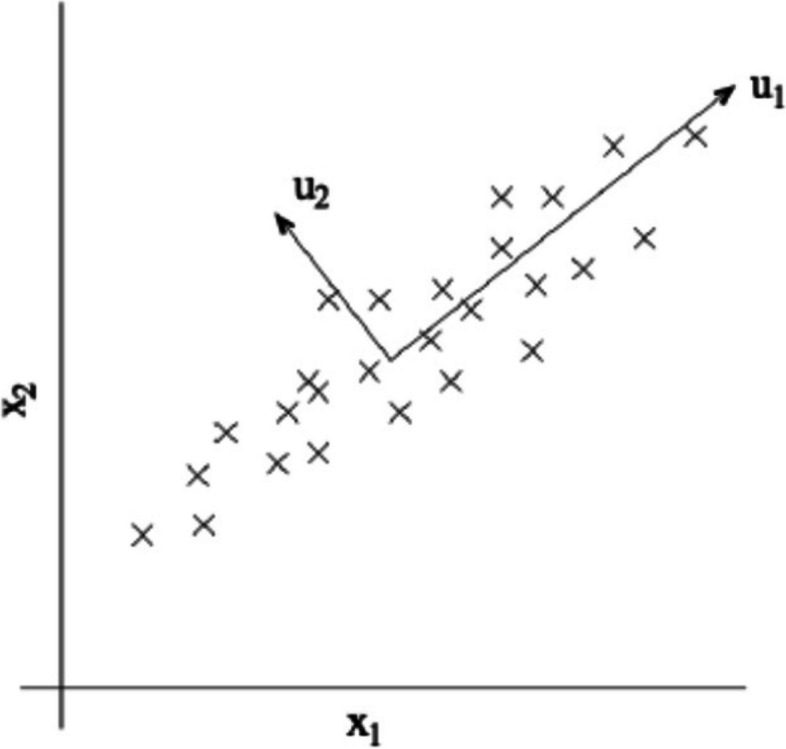
Data Projection in subspace

The common goal of PCA is dimensionality reduction. x is an eigenvector whose covariance matrix is X, then the result of extracted features with respect to x, of an random sample vector v is calculated using the Eq. (1)

1 $$\:\text{y}\:=\:{v}^{T}x\:=\:\sum\:_{i=1}^{D}{v}_{i}{x}_{i}$$

where, $\:x={\left[{x}_{1}\dots\:{x}_{D}\right]}^{T}$, $\:v={\left[{v}_{1}\dots\:{v}_{D}\right]}^{T}$ and D is the dimensionality of the sample vectors. The absolute value of $\:{x}_{i}\left(i=\text{1,2},\dots\:.,D\right)$ is capable of evaluating the result of feature extraction of the $\:{i}^{th}$ feature component of all the samples.It is very simple to find the smaller absolute value of $\:{x}_{i}$ and the lesscontribution of the $\:{i}^{th}$ feature element of sample data. Do removing $\:{v}_{k}{x}_{k\:}$ from $\:\sum\:_{i=1}^{D}{v}_{i}{x}_{i}$ sincethe absolute value of $\:{x}_{s}$ is too little. It will not consider to the effect of feature extraction result. It is essential to add the feature component which seems to be important in feature extraction in the original subspace. If any of the feature components absolute value seems to be small then remove it by considering it as not important. There are always multiple eigenvector involved in the process of assessing the consequence of the useful component. The algorithm 1 discusses the process of feature extraction:

**Figure:**
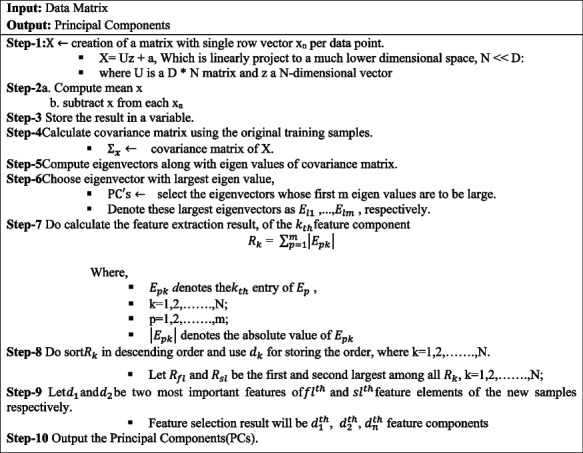
**Algorithm 1: **PCA

Figure 2 illustrates the data projection in 2-dimensional subspace. From the data extraction the model arrives with the detailed information about medical features of patients, study plan, medical transcriptions, DR assessment and the total number of patients who has symptoms of CVD based on DR status. The numeric data mentioned in Table 1 are used in this work. In the few works these data are not reported and the risk factors are determined from the continued existence curves. The output of this method produces the useful features for the feature selection phase by removing the outliers and irrelevant features from the raw data.

### Particle swarm optimization

I-DBN uses PSO an optimum based algorithm for selecting the suitable features among the features extracted. The extracted features from the PCA are given as input to this method. This feature selection method is useful for the model to enhance the accuracy of the prediction with less effort in both the training and testing end. PSO is a stochastic optimization technique which uses iterative method. PSO inspired based on the observation of social behavior of bird flocking. This algorithm searches for optimal solution by initializing a population with random solution and by updating generations. All the potential solutions are considered as particles [43]. Each particle retains its coordinates in the search space. The population of PSO is called a swarm. A cornfield model is developed by Heppner to replicate the searching behavior of a flock of birds [44]. At the beginning of the search, the location of food and birds are arbitrarily dispersed in the searching plane. The birds have to move with certain rules to find the food’s location. The following are the coordinates associated with the cornfield mode:

(x_0_, y_0_) – denotes the position of the cornfield.

(x, y) – denotes the position coordinate of an individual bird.

(v_x_, v_y_ ) – denotes the velocity coordinate of an individual bird.

The current position and model’s speed is measured by the distance among the current position and cornfield [45]. The above mentioned coordinates are used to calculate the function f_k_, where the random position of the particles is denoted as $\:\overrightarrow{\text{p}\text{p}}$ and velocity of the particles is $\:\overrightarrow{\:\text{v}}$. At each time interval the particle Positions and velocity coordinates are adjusted to the new coordinates and the function is evaluated. If any particle founds new pattern then the corresponding coordinates are stored in a vector [44]. Then the particle velocities and particle positions are updated by using the below mentioned Eqs. (2)&(3)

All particle velocities will be updated using the Eq. (2),

2 $$v^{i}_{x+1}=\omega\,v^{i}_{x}+s_{1}r_{1}(P^{i}_{x}-pp^{i}_{x})+s_{2}r_{2}(P^{k}_{x}-pp^{i}_{x})$$

3 $$\:{pp}_{x+1}^{i}\:=\:{pp}_{x}^{i}\:+\:{v}_{x+1}^{i}$$

All particle positions will be updated using the Eq. (3),

In the Eqs. (2) and (3).

- $\:{v}_{x+1}$ - denotes the velocity
- $\:\omega\:$- denotes inertia weight.
- Both of these notations are used to make the global and local exploitation to be a scalable one.
- r_1_ and r_2_– denotes are random variables that distributed uniformly with range [0, 1].
- s_1_ and s_2_– denotes acceleration coefficients.

The upper bound has to be set to the velocity parameter. A new way called Velocity clamping [46] was used to limit the swarms flying out of the two dimensional space. Another strategy called Constriction Coefficient [47] used as an analysis approach of swarm dynamic. By using this method the velocities are constricted too. First half of the Eq. (2), known as inertia, which denotes the previous velocity.

It provides the needed momentum for swarms to move into the search space. The next half of the Eq. (2) represents the cognitive component through which the individual particles are thinking of each swarms. It makes the particles to travel on the way to their own best point. The third part of the Eq. (2) denotes the cooperation element. It uses the mutual effect of the swarms to determine the best finest solution [48]. The cooperation component also share the information about particle’s moves come from other particles’ knowledge in the swarm. By using cognition technique, the movement of the particles is stimulated. The parameter S_2_ is known as social acceleration factor. Its natural background made the implementation simple and easy and also the wide adaptability to different types of functions. Because of these reasons the PSO algorithm has been used in the I-DBN model.

The difference among the best position and the individual’s present position is added to the present velocity which causes the curve fluctuations around that point. The movement of particles in search space helps in identifying the two best positions.

Two fitness values calculated as follows:

- The PP-fitness and P-fitness records its corresponding PP- Particles Position vector and P vector respectively.
- The particles will be treated as simple agent. This can fly through feature space and record the best solution that they have discarded.
- In two dimensional search space, the particle moves from one space to another. The location transformation happens by adding T-vector to the CP-vector to get another CP-vector.

4 $$\overrightarrow{pp_{l}}=\overrightarrow{pp_{l}}+\overrightarrow{T_{l}}$$

Where, the T -vector T = < T_k0_,T_k1_,T_k2_,…T_kn-1_> records about a gradient to make a particle to travel in V.

The algorithm 2 discusses about feature selection,

**Figure:**
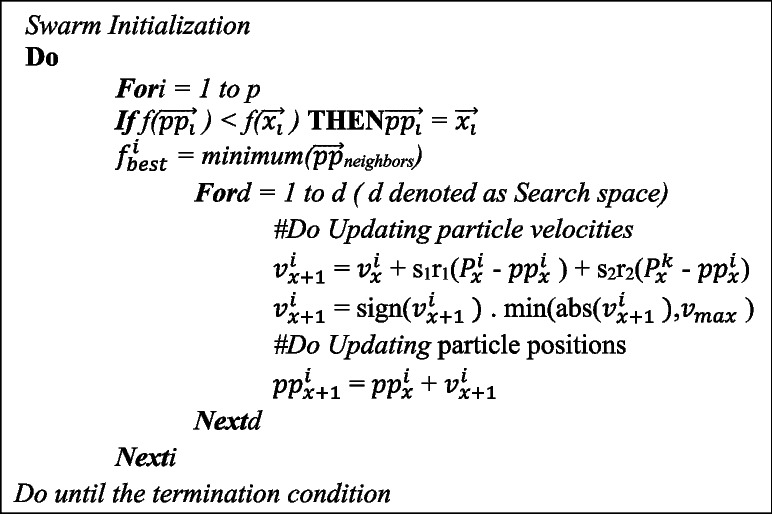
**Algorithm 2**: PSO

$\:\overrightarrow{{pp}_{i}}$ will be calculated to evaluate particles new location. Based on the betterment of fitness between PP –fitness and P-fitness equalize $\:\overrightarrow{{x}_{i}}$ = $\:\overrightarrow{{pp}_{i}}$ and $\:\overrightarrow{{x}_{i}}$ fitness=$\:\overrightarrow{{pp}_{i}}$ fitness.

The pbest value is calculated using Eq. (5)

5 $$pbest(i,\,t)=\:{\text{a}\text{r}\text{g}}_{(\text{k}=1,\dots\:,\text{t})}\text{m}\text{i}\text{n}\left[\text{f}\left(\overrightarrow{{x}_{i}}\left(\text{k}\right)\right)\right], Where\,i{\epsilon}\left\{1,2,...N_{p}\right\}$$

The gbest value is calculated using Eq. (6)

6 $$gbest(t)=\:{\text{a}\text{r}\text{g}}_{(\text{k}=1,\dots\:,\text{t})}{\text{m}\text{i}\text{n}}_{\text{i}=1,\dots\:{\text{N}}_{\text{p}}}\left[\text{f}\left(\overrightarrow{{x}_{i}}\left(\text{k}\right)\right)\right]$$

PSO focused on searching a better location for particles through several iterations. In this regard, particle’s information is updated from first iteration into the next. To obtain the optimal solution, each particle moves towards the pbest and gbest position in the swarm. The particles’ best position is calculated by the above mentioned Eqs. (5) and (6). The best and finest selected features are given as input to our I-DBN. By giving the suitable optimized features into the model, we may reduce the training and testing load of the I-DBN model. It also brings the model in resulting with less validation loss.

### Deep nelief network

In this work I-DBN model is proposed to classify the DR dataset in order to know the risk level of causing CVD. The best particles’ positions calculated from the above PSO are given as input to this network. These finest particles are makes DBN to train its network with optimized one. With a variety of deep learning models being available, DBNs has been played an important role in all kinds of practical applications [49–51]. DBN is the first non-convolutional model which successfully confesses the training. It a kind of DNN composed of multi-layer Restricted Boltzmann Machine (RBMs). RBN is a significant element of DBN used for classification task [52, 53]. RBN is an undirected graphical model includes two layers like a visible layer and a hidden layer of binary units [54]. The visible layer relates the visible features where the hidden layer represents high-level features of the input data. There is an undirected connection between the layers of an RBN while there are connectionless nodes in the same layer of the network model.

**Fig. 3:**
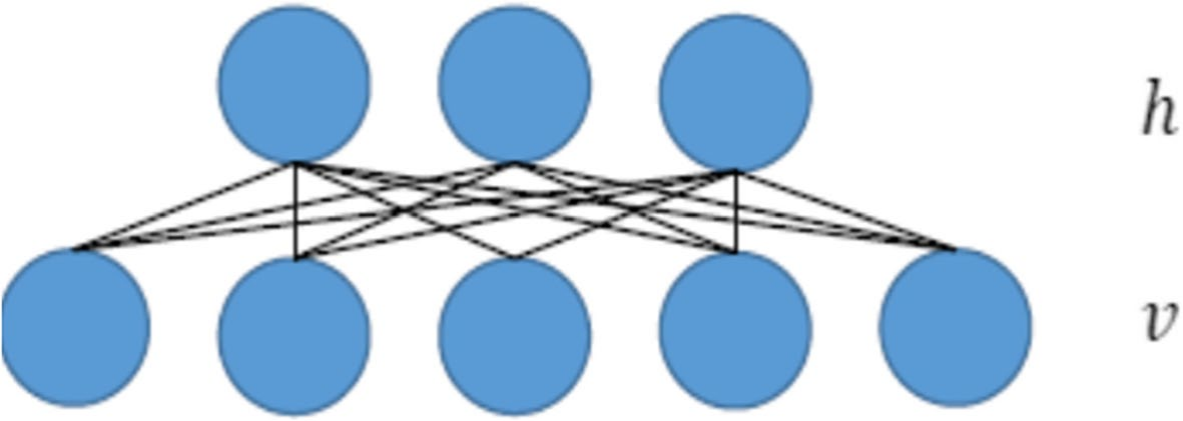
Schematic diagram of RBMs

**Fig. 4:**
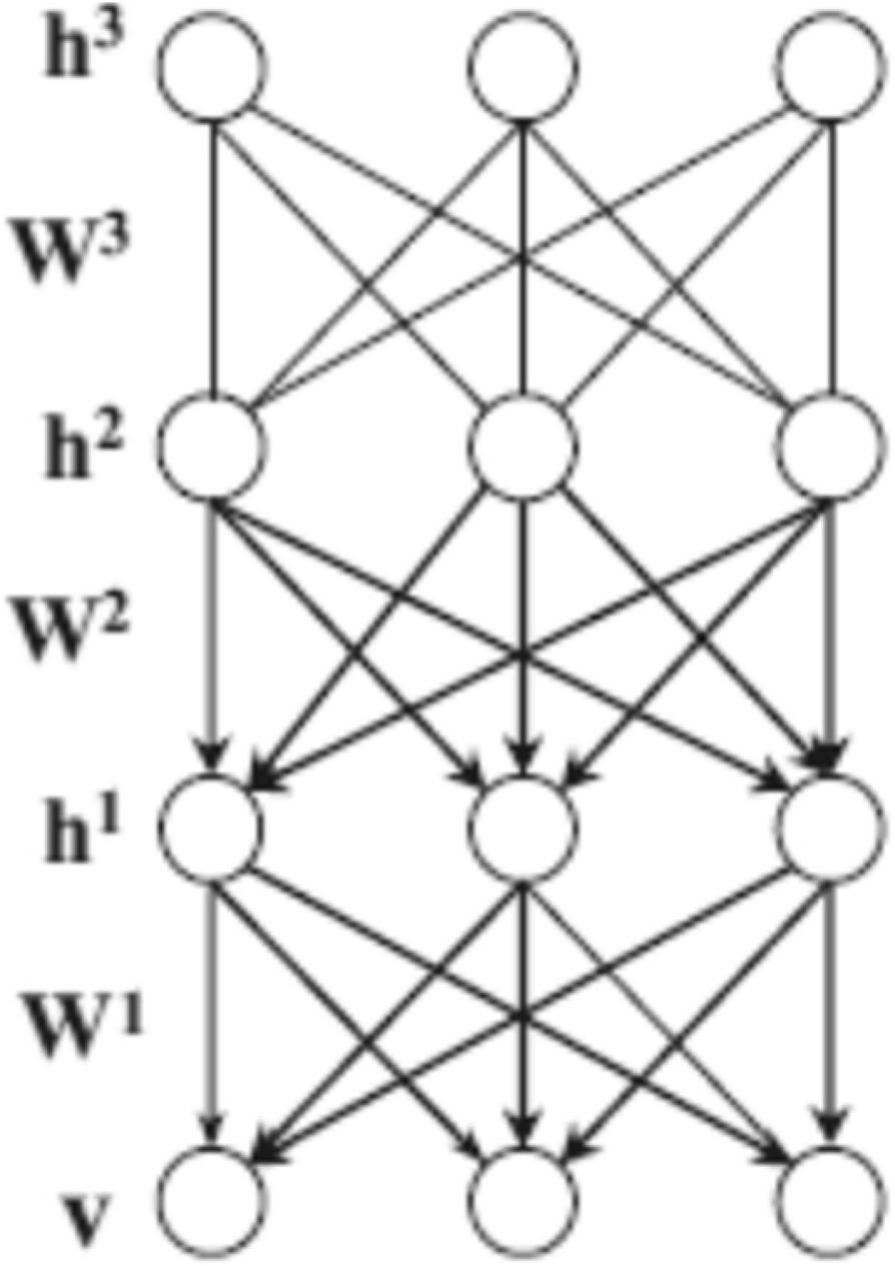
Schematic diagram of DBN with three hidden layers

The Fig. 3 shows the schematic diagram of RBN and DBN. It represents the undirected bipartite model with connections between visible (v) and hidden (h) nodes. RBN is used to extract the features are transmitted to the upper layer of RBM. The extracted features from the last layer of RBM are transferred to the Back Propagation (BP) neural network. Multi-layers’ structural description make simpler in obtaining the compression coding of the dataset. When the neural network uses the RBN at the first time, the energy function has been initiated with 2 vectors namely $\:{v}_{n}$,$\:{h}_{n}$.$\:{v}_{n}$and $\:{h}_{n}$ denotes neurons or unit of visible and hidden layers respectively. Figure 4 illustrates about DBN with three hidden layers.

Corresponds to the joint probability distribution,

7 $$P(v_{n}, h_{n})=\frac{1}{z}e^{-energy(v_{n}, h_{n})}$$

8 $$Z=\sum\nolimits_{v_{n},h_{n}}e^{-energy(v_{n},h_{n})}$$

Where, in Eq. (8), $\:-energy\left({v}_{n},{h}_{n}\right)$ is the energy function which is defined as,

9 $$\:-energy\left({v}_{n},{h}_{n}\right)\:=\:\left({{v}_{n}}^{{\prime\:}}W{{h}_{n}}^{{\prime\:}}+{b}^{{\prime\:}}{v}_{n}+{c}^{{\prime\:}}{h}_{n}\right)$$

In the Eq. (9) {W, b,c} are parameters and *Z* is a normalization factor of $\:P\left({v}_{n},{h}_{n}\right)$. The effects of the latent identifiers are accepted by allowing them for the marginal distribution above the visible units:

10 $$\:P\left({v}_{n};{\Theta\:}\right)=\:\sum\limits_{h}\frac{1}{Z\left(\varTheta\:\right)}{e}^{-energy\left\{\left({v}_{n},{h}_{n};{\Theta\:}\right)\right\}}$$

The expectation with respect to the model distribution is defined with the help of the Eq. (10).

To compute marginal $\:\text{P}\left(\text{x}\right)$

11 $$\begin{aligned} P(V_{n}) &=\sum_{h_{n}\epsilon\left\{0,1\right\}^{H}}e\frac{(v_{n}^{'}Wh_{n}^{'}+b^{'}\,v_{n}+c^{'}h_{n})}{Z} \\ &=e^{\left(c^{'\:}v_n\right)}\sum_{{}_{h_{n\:}\in\:\left\{\text{0,1}\right\}^H}}e^\frac{\left(v_n^{'\:}Wh_n^{'\:}+b^{'\:}v_n+c^{'\:}h_n\right)}Z\\ &=\:exp\left(c^{'\:}v_n\right)\:exp\left(log\left(1+exp\left(b_1+W_1.v_n\right)\right)\right)\dots\:exp\left(log\left(1+exp\left(b_H+W_H\:.\:v_n\right)\right)\right)/Z \end{aligned}$$

12 $$P\left(v_n\right)=\:exp\left(c^{'\:}v_n+\sum\limits_{j=1}^Hlog\left(1+exp\left(b_j+W_jv_n\right)\right)\right)/Z$$

The above Eq. (13) is also known as Product of Experts model.

13 $$\:P\left(v_n\right)=\:exp\left(c^{'\:}v_n+\sum_{j=1}^Hlog\left(1+exp\left(b_j+W_jv_n\right)\right)\right)/Z$$

14 $$\:\:\:\:\:P\left(v_n\right)=\:exp\left(c^{'\:}v_n+\sum_{j=1}^Hlog\left(\text{s}\text{o}\text{f}\text{t}\text{p}\text{l}\text{u}\text{s}\left(b_j+W_jv_n\right)\right)\right)/Z$$

In the Eq. (15) where,

$\:{c}^{{\prime\:}}{v}_{n}$ - bias the probability of each $\:{x}_{i}$

$\:{b}_{j}$*-* bias of each feature.

$\:{W}_{j}{v}_{n}$-feature expected in x

From this derivation the model arrives with the marginal probability distribution of P(x).

### RBN Model Learning

It is must to maximize the marginal likelihood of $\:{v}_{n}$ toestimate $\:\theta\:$, in an unsupervised manner i.e.,

15 $$\:{P}_{\theta\:}\left({v}_{n}\right)=\:\frac{{P}^{*}\left({v}_{n}\right)}{Z\left(\theta\:\right)}=\:\frac{1}{Z\left(\theta\:\right)}\sum_{{h}_{n}}exp\left[{{v}_{n}}^{{\prime\:}}W{h}_{n\:}+{a}^{{\prime\:}}{h}_{n}+{b}^{{\prime\:}}{v}_{n}\right]$$

Given a group of independent and identical distribution of training examples D=$\:\left\{{{v}_{n}}^{\left(1\right)}\:,\:{{v}_{n}}^{\left(2\right)}\:,\dots\:,\:{{v}_{n}}^{\left(N\right)}\right\}$. Then, we need to make learn the model parameters,$\:\theta\:$=$\:\left\{W,a,b\right\}.$ Maximization of log-likelihood objective is happened by using the Eq. (17).

16 $$\:L\left(\theta\:\right)=\:\frac1N\sum_{n=1}^N\text{log}P_{\theta\:}\left(v_n^{\left(n\right)}\right)$$

Stochastic Gradient Ascent approach is used commonly to maximize log-likelihood i.e., $\:\text{log}P\left({v}_{n};\theta\:\right)$. We have to estimate the gradient oflog-likelihood with respect to θ, repeatedly, the log-likelihood is calculated over all observed data.

Thus derivative of the log-likelihood will be calculated using Eq. (18)

17 $$\:\frac{\partial\:L\left(\theta\:\right)}{\partial\:{W}_{ij}}=\:\frac{1}{N}\sum_{n=1}^{N}\frac{\partial\:}{\partial\:{W}_{ij}}\:log\left(\sum_{h}exp\left[{{v}_{n}}^{\left({n}^{{\prime\:}}\right)}W{h}_{n\:\:}+{a}^{{\prime\:}}{h}_{n}+{b}^{{\prime\:}}{{v}_{n}}^{\left(n\right)}\right]\right)-\:\frac{\partial\:}{\partial\:{W}_{ij}}\text{log}Z\left(\theta\:\right)$$

Based on $\:{{v}_{n}}^{\left(0\right)}$, its corresponding gradient is decomposed into two phases. One is positive phase the other one is negative phase.

18 $$\begin{aligned}\:\frac{\partial\:}{\partial\:\theta\:}\text{log}P\left({{v}_{n}}^{n}\right)=\:\frac{\partial\:}{\partial\:\theta\:}\:log\:\sum\:_{h}exp\left({{v}_{n}}^{{n}^{{\prime\:}}}W{h}_{n}+{c}^{{\prime\:}}{{v}_{n}}^{\left(n\right)}+{b}^{{\prime\:}}{h}_{n}\right)-\frac{\partial\:}{\partial\:\theta\:}\text{log}Z\\Positive\,Phase\,Negative\,Phase\end{aligned}$$

The second term in Eq. (18) is a negative phase which is of intractable due to exponential number of configurations which is denoted in Eq. (19).

19 $$\:Z=\sum\:_{{v}_{n}}\sum\:_{{h}_{n}}exp\left({{v}_{n}}^{{\prime\:}}W{h}_{n}+{c}^{{\prime\:}}{v}_{n}+{b}^{{\prime\:}}{h}_{n}\right)$$

The first term in Eq. (18) is positive phase which denoted in Eqs. (20),

20 $$\frac{\partial}{\partial{\theta}}log\sum_h{exp}(v_{n}^{n^{\prime}}Wh_{n}+c^{\prime}v_{n}^{(n)}+b^{\prime}h_{n})=E_{h\sim{p}(v^{n},h)}[h_{n}v_{n}^{(n)^{\prime}}]$$

The maximization log-likelihood $\:\text{l}\text{o}\text{g}\:\text{p}\left({\text{v}}_{\text{n}},{\uptheta\:}\right)$ process with respect to $\:{\uptheta\:}=\{\text{b},\text{W},\text{c}\}$ is done using Eqs. (21), (22),

21 $$\:\frac{\partial\:}{\partial\:{W}_{ij}}\text{log}P\left({{v}_{n}}^{\left(n\right)}\right)={energy}_{{\:h}_{nj}}\left[{v}_{ni}{h}_{nj\:}|{v}_{n}={{v}_{n}}^{\left(n\right)}\right]-{energy}_{{v}_{ni},{h}_{nj\:}}\left[{v}_{ni}{h}_{nj\:}\right]$$

22 $$\:\frac{\partial\:}{\partial\:{b}_{j}}\text{log}P\left({{v}_{n}}^{\left(n\right)}\right)={energy}_{{\:h}_{nj}}\left[{h}_{nj\:}|{v}_{n}={{v}_{n}}^{\left(n\right)}\right]-{energy}_{{h}_{nj\:}}\left[{h}_{nj\:}\right]$$

23 $$\:\frac{\partial\:}{\partial\:{c}_{i}}\text{log}P\left({{v}_{n}}^{\left(n\right)}\right)={energy}_{{\:v}_{ni}}\left[{v}_{i\:}|{v}_{n}={{v}_{n}}^{\left(n\right)}\right]-{energy}_{{v}_{i\:}}\left[{v}_{i\:}\right]$$

In the Eq. (23) the term, $\:energy\left[{v}_{i}|{v}_{n}={{v}_{n}}^{\left(n\right)}\right]$ is represented as positive statistic.

$$\:energy\left[{v}_{i}|{v}_{n}={{v}_{n}}^{\left(n\right)}\right]=\:energy\left[{v}_{i}|{v}_{n}={{v}_{n}}^{\left(n\right)}\right]{{v}_{ni}}^{\left(n\right)}$$

Ideally, we have integrated to compute $\:energy\left[{v}_{ni}{h}_{nj}\right]$ (negative statistic). However, to estimate the gradients a sampler over time can be used. In the approximate learning, by replacing the overall average possible sample input configurations. It has been done using the Eqs. (24),

24 $$\:{energy}_{{v}_{n},{h}_{n}}=\:\sum_{{h}_{n}\:,{v}_{n}}p\left({h}_{n},{v}_{n}\right){h}_{n}{{v}_{n}}^{{\prime\:}}$$

We have to do running Gibbs sampling technique starts from the observed samples. From the Eq. (25), $\:P\left({h}_{n}\:,\:{v}_{n0}\right)$ is represented as the conditional distribution when given $\:{v}_{n0\:}$.

25 $$\:P\left({h}_{n}\:,\:{v}_{n0}\right)=\frac{{e}^{\left(-energy\left({v}_{n0},{h}_{n}\right)\right)}}{{\sum\:}_{h}{e}^{\left(-energy\left({v}_{n0},{h}_{n}\right)\right)}}$$

It is clear that from Eq. (18), the gradient maximizes the log-likelihood which is equals to the difference among model’s expectation and data distributions [55, 56]. The second phase of the gradient is intractable, so we have to use sampling methods for approximating its value. Because of the unique characteristic of RBM connectionless layer units are not dependant with each other in $\:{h}_{n}$, when the condition on $\:{v}_{n}$, and vice versa. It brings the result as follows,

26 $$\:p\left({v}_{n}|{h}_{n}\right)=\prod\:p\left({v}_{ni}|{h}_{n}\right)$$

27 $$\:p\left({h}_{n}|{v}_{n}\right)=\prod\:_{j}^{i}p\left({h}_{nj}|{v}_{n}\right)$$

28 $$\:p\left({v}_{ni}=1|{h}_{n}\right)=\sigma\:\left({{W}_{i}}^{{\prime\:}}{h}_{n}+{b}_{i}\right)$$

29 $$\:p\left({h}_{nj}=1|{v}_{n}\right)=\sigma\:\left({W}_{j}{v}_{n}+{c}_{j}\right)$$

Where $\:\sigma\:\left(x\right)$ is known as logistic sigmoid function.It can be written as$\:\sigma\:\left(x\right)={\left(1+{e}^{-x}\right)}^{-1}$. By using this sigmoid function the second term in Eq. (18) can be computed easily. To find the gradient mentioned the first term in Eq. (18) is the leading challenge to sample from the distribution of the model $\:P\left({v}_{n},{h}_{n}\right)$.

Getting an unbiased sample of the second term is very tough. Generally, many steps need to be iterated to reach the equilibrium for getting approximate gradient value of $\:\text{p}\left({\text{v}}_{\text{n}}|{\text{h}}_{\text{n}}\right)$. It can be solved by performing Gibbs Markov Chain Monte Carlo (MCMC) method at any random state of the visible units using Eqs. (26), (27). This Gibbs MCMC algorithm is used to run $\:\text{p}\left({\text{h}}_{\text{n}}|{\text{v}}_{\text{n}}\right)$ and $\:\text{p}\left({\text{v}}_{\text{n}}|{\text{h}}_{\text{n}}\right)$ one after another. Contrastive Divergence (CD) method is used to provide a realistic value of the gradient. The purpose of using CD is to make run the Gibbs chain for only times (steps) to generate approximate samples. This CD algorithm facilitates in estimating the gradient easily. This also helps in speeding the training phase of RBMs as well and it sets off the repetition of Neural Networks [46]. The algorithm 3 discusses about the generation of approximate samples.

**Figure:**
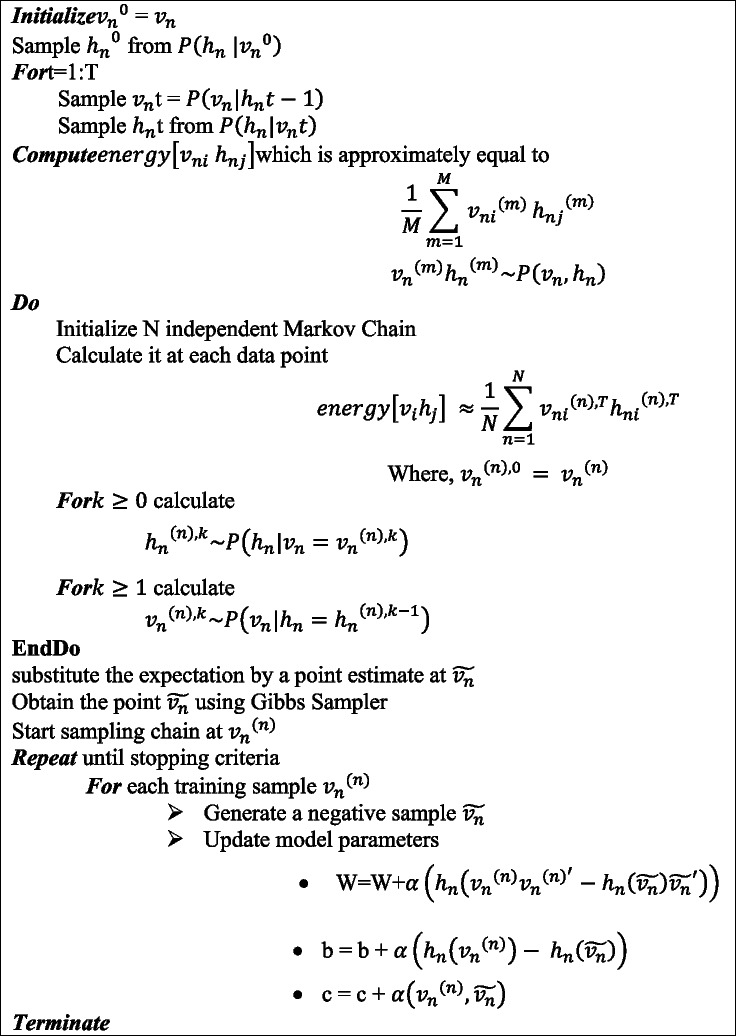
**Algorithm 3:** Block-Gibbs MCMC Algorithm

In order to assess the prognostic value of DR the model needs to find the Odds Ratio (OR). This ratio helps in analyzing all cause cardiac mortality and risk events. DR is estimated either as basic degree or complex degrees among the cluster with no symptoms of DR in analyses classified by it types. This model evaluates the risk by obtaining the random effects to the heterogeneity of various studies. The significant threshold value for the model is set to be *P* = 0.10. Regression based analyses are applied to examine the latent sources. An average proportion among positive and negative cases is determined to attain the approximate calculation of sensitivity and specificity.

## Experimental setup

I-DBN uses Diabetic Retinopathy (DR) Debrecen Data Set from UCI Machine Learning Repositories for the experimental results. The dataset has 1151 instances with 20 attributes. The description of the attributes is mentioned in Table 1. Table 2 depicts the total number of instances taken for training and testing the proposed model bases the class 0 and 1. Out of 1151 instances from 20 attributes, there are 560 instances of class_0 and 591 instances of class_1 were included for both training and testing the model respectively.

**Table 2:** Summary of training and testing samples

Attribute @20	Training Samples	Testing Samples	Total number of instances
**Class_0**	443	117	560
**Class_1**	461	130	591

The features of these dataset are extracted from the Messidor image set. All features represent either a detected lesion, a descriptive feature of an anatomical part or an image-level descriptor. A Personal Computer contains 8 GB RAM used to perform the experiment. Python tool is used for program execution.

## Result and discussions

### Performance metrics for model evaluation

The metrics mentioned below are used to assess the I-DBN model.

Accuracy:

It is the ability to differentiate the disease and non-disease correctly. Also, it is the ratio of correctly classified prediction to the total number of predictions. It can be determined using the Eq. (30)

30 $$\mathrm{Accuracy}=\:\frac{\left(TN+TP\right)}{\left(TN+TP+FN+FP\right)}$$

Where, TP means True Positives, TN means False Negatives, FP represents False Positives, FN meant to be False Negatives.

#### Sensitivity

It is a metric used to determine the disease correctly. It is calculated as the proportion of TP in disease cases. It is represented as Eq. (31)

31 $$\mathrm{Sensitivity}=\:\frac{TP}{\left(TP+FN\right)}$$

#### Specificity

It is a metric used to determine the healthy persons correctly. It is calculated as the proportion of TN in normal healthy people. This is stated as Eq. (32)

32 $$\mathrm{Specificity}=\:\frac{TN}{\left(TN+FP\right)}$$

#### Precision

It computes the number of positive class predictions that are actually belonging to the positive class. It can be calculated using Eq. (33)

33 $$\mathrm{Precision}=\:\frac{TP}{TP+FP}$$

#### Recall

It is defined as the number of TP made out of all positive cases in the dataset. It can be estimated by using Eq. (34)

34 $$\mathrm{Recall}=\:\frac{TP}{TP+FN}$$

### Performance analysis

I-DBN is evaluated using a stochastic gradient-based model. This model was used to build the PSO-DBN model. To perform the cross-validation process, the dataset was divided into two; one for training the model and another is for testing the proposed model. In the dataset 80% were given for training phase and 20% were used for validating/testing the model with each and every 64 records (batchsize).

The proposed I-DBN model is composed of stacked RBNs. The stochastic gradient ascent algorithm is used for RBN model learning. Logistic sigmoid function is applied to find the negative phase of the log-likelihood. Gibbs Markov method helps to generate approximate samples [41, 42, 48]. CD algorithm is chosen for estimating the gradients easily and also it speeds up the training of RBN [43]. Total number of epochs used in the I-DBN model is 75. The learning rate of the model is 0.12. Table 3 illustrates the overall outline of the results with 2269 record of the bench DR dataset.

The hyperparameters for the I-DBN model are carefully selected to balance model performance, computational efficiency, and predictive stability, guided by iterative testing and alignment with the model’s objectives. The batch size was set to 64 records, chosen to balance memory usage and ensure stable training within the stochastic gradient-based model. This study used an 80/20 data split to provide ample data for learning while retaining a substantial portion for testing, enhancing the model’s robustness. The I-DBN model comprises stacked RBMs optimized through Particle Swarm Optimization (PSO) to capture intricate patterns for CVD prediction, structured as a deep belief network for feature selection efficiency. A learning rate of 0.12 is selected after trials showed it facilitated stable convergence without excessive weight fluctuations, helping prevent overfitting. The model is trained for 75 epochs, a value chosen based on the observed convergence pattern, ensuring that the model fully utilized the training data without further performance gains beyond this point. To further improve training efficiency, the CD algorithm is used for gradient estimation, which simplified the calculations required by the stacked RBMs. Gibbs Markov sampling was applied for generating approximate samples, as this method supports stable training while capturing data distribution effectively. These hyperparameter choices provide a transparent framework that aligns with the I-DBN model’s goals of minimizing training time, reducing overfitting, and achieving high reliability in CVD prediction.

**Table 3:** Outline of the experimental results with 2269 records

Method	Accuracy	Precision	Recall	Sensitivity	Specificity
DNN	95.78	94.56	94.6	90.4	93.87
DNN + PCA	90.07	90	91	87	91
SVM	73.6	74.82	73.68	83.5	83.55
SVM + PCA	65.3	78	65	85	82
DT	88.2	89	89	85.6	90.9
DT + PCA	86	86	87	84	89
KNN	58.2	58	58	59.8	56.6
KNN + PCA	64	64	65	63	62
NB	57	57	59	60	57
NB + PCA	63.5	65	64	62	64
XGBoost	83.3	84	83	75.2	63.8
XGBoost + PCA	80	80	80	81	79
**DBN**	**93.95**	**93.9**	**95.11**	**93.9**	**94.17**
**Improvised DBN (I-DBN)**	**98.95**	**97.61**	**98.78**	**95.89**	**97.87**

The primary contribution of this study is cantered on the optimization of hyperparameters and network design within the I-DBN model, achieved through the application of Particle Swarm Optimization (PSO). PSO enhances the model’s predictive capability by selecting optimal hyperparameters, which improves accuracy, efficiency, and overall robustness. This optimization is further validated by comparing the I-DBN model’s performance against multiple state-of-the-art classifiers (DNN, SVM, DT, KNN, NB, and XGBoost), both with and without the application of Principal Component Analysis (PCA).

While PCA is used as a dimensionality reduction technique to reduce computational load and test the model’s resilience under reduced feature dimensions, it is not a feature selection method in this study. Instead, PCA’s inclusion allows us to examine the consistency of I-DBN’s performance when dimensionality is minimized. This approach demonstrates that the I-DBN model remains robust even after applying PCA, thereby emphasizing that the model’s effectiveness is primarily due to the hyperparameter optimization achieved through PSO.

Table 3 demonstrates the performance evaluation of these classifiers with randomly increased dataset. The state-of-the-art classifiers performances are compared with proposed I-DBN. It is also identified that the performance of I-DBN is better than other ML algorithms. It shows the experimental results obtained using the I-DBN model against various other network models. I-DBN approach gives better results than the others approach about accuracy 93.95%, precision of 93.9%, recall of 95.11%, sensitivity of 93.9% and specificity of 94.17%. It shows the performance assessment of the various classifiers with the original records (1151) of the dataset and randomly increased dataset. DBN + PCA have been used on the augmented dataset. It is noted that the performance of DBN with real dataset is higher than the other models with respect to all the metrics. The performance of DBN has improved further when the size of the dataset is augmented up to 2269 records. In-order to achieve these results PCA has been applied to the augmented dataset. The accuracy value of DBN has improved about 98.95% when combined with PCA. DBN + PCA bring out precision of 97.61%, recall of 98.78%, sensitivity of 95.8% and specificity of 97.87%. Also, it is observed from these figures that the performance of DBN has not been affected to a big deal even after applying the feature reduction technique [44, 47]. PSO is used for selecting the suitable parameters to the DBN. The proposed model outperformed with other models compared in this study.

**Fig. 5:**
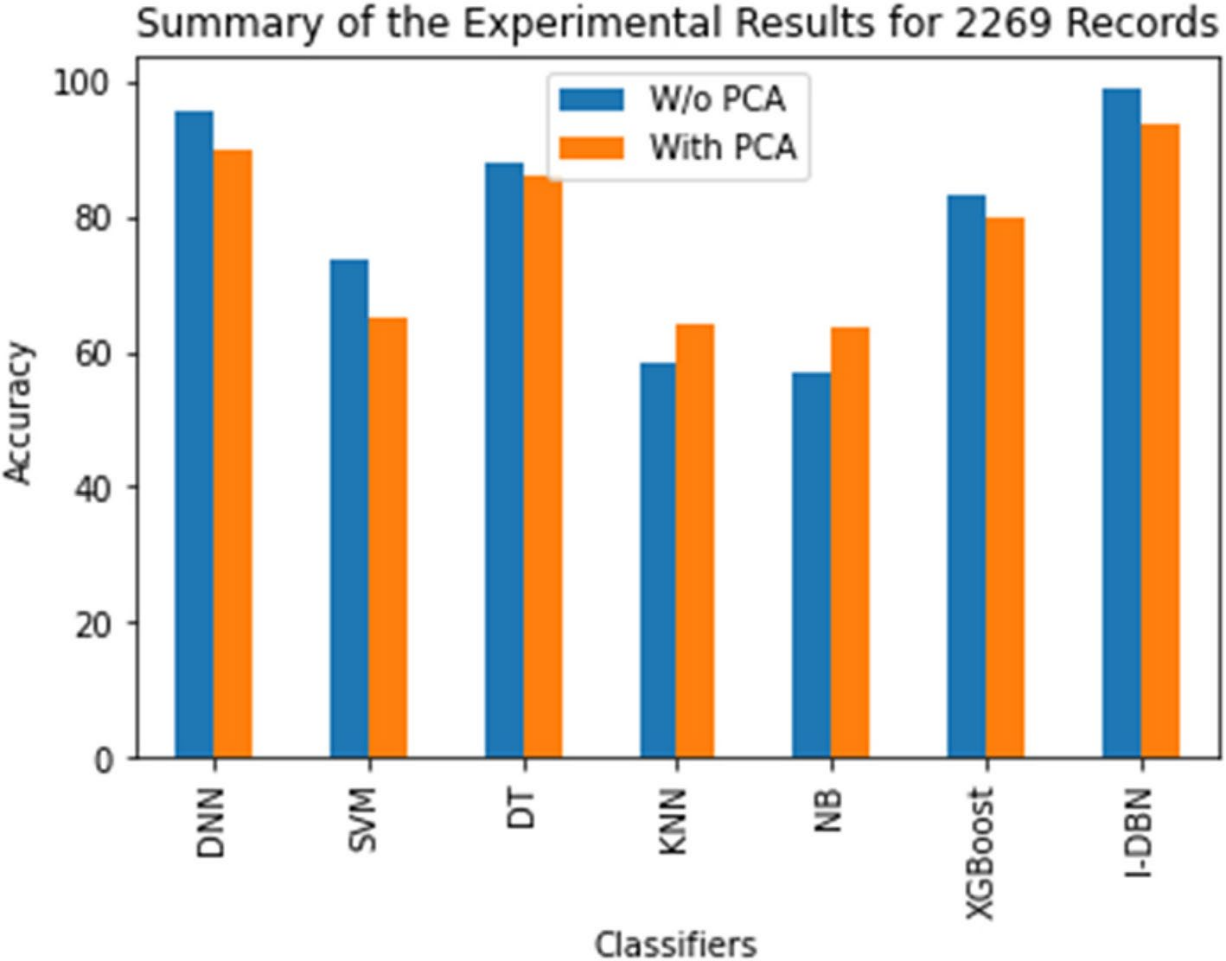
Performance Evaluation of I-DBN with other classifiers on accuracy

**Fig. 6:**
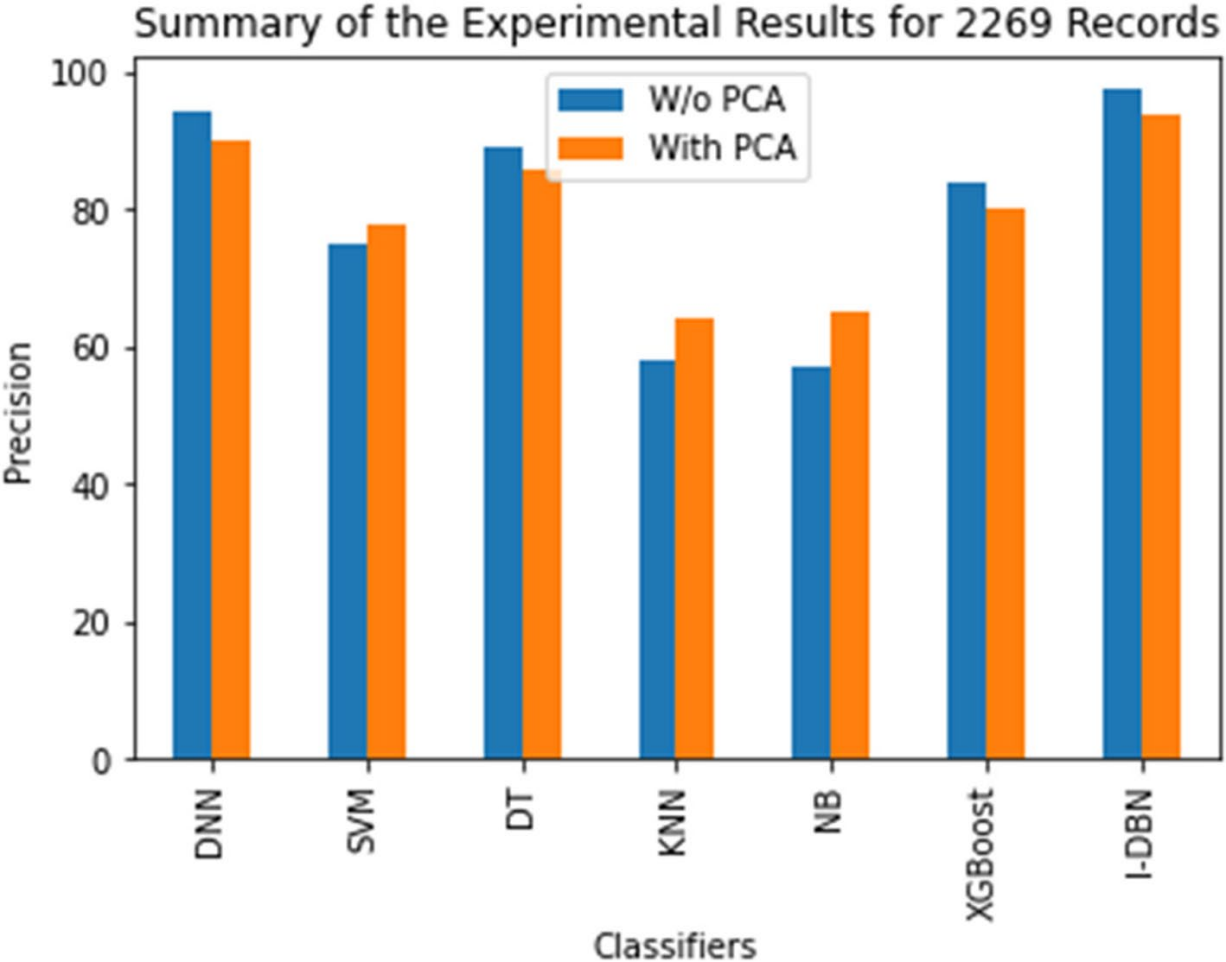
Performance Evaluation of I-DBN with other classifiers on precision

**Fig. 7:**
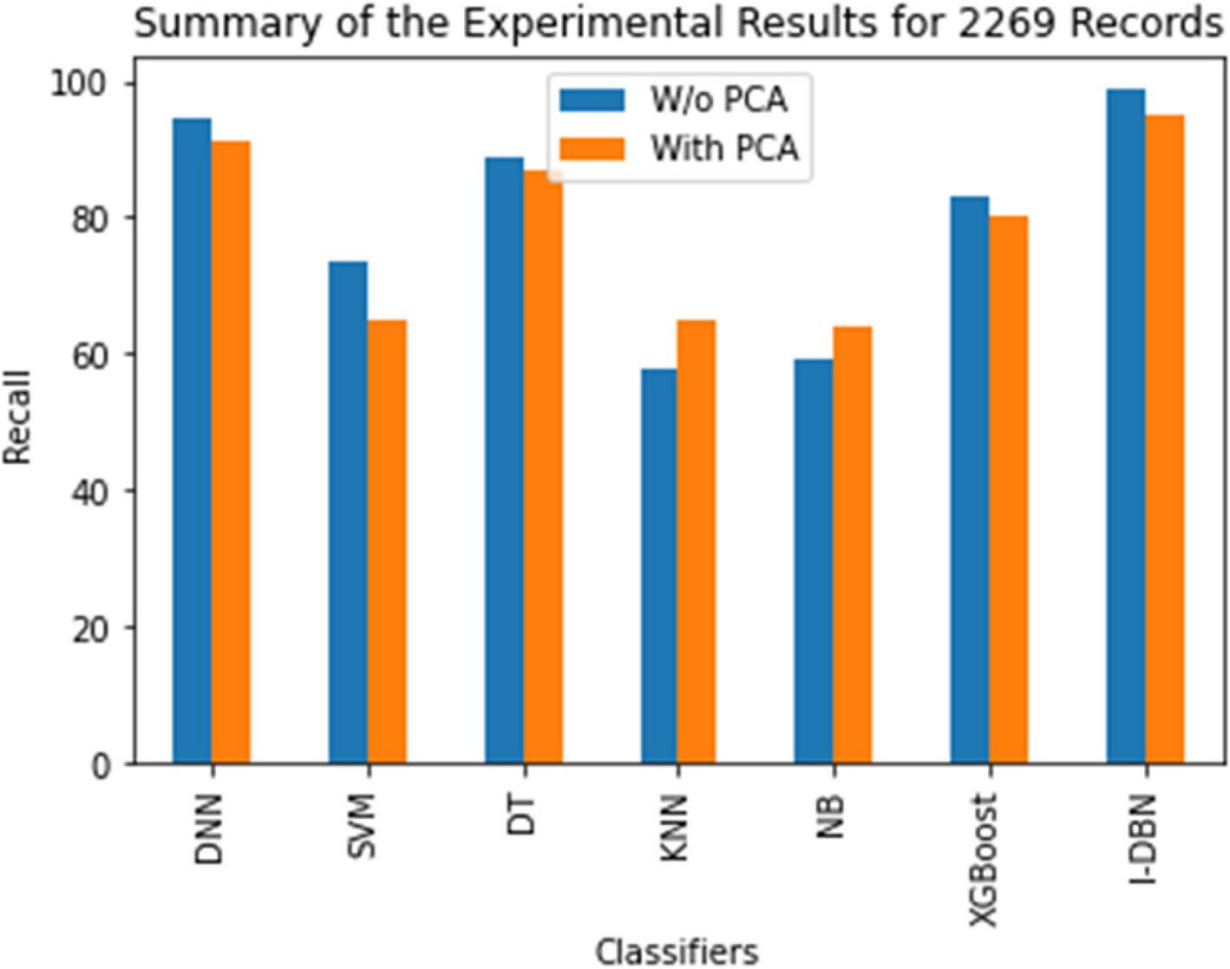
Performance Evaluation of I-DBN with other classifiers on recall

**Fig. 8:**
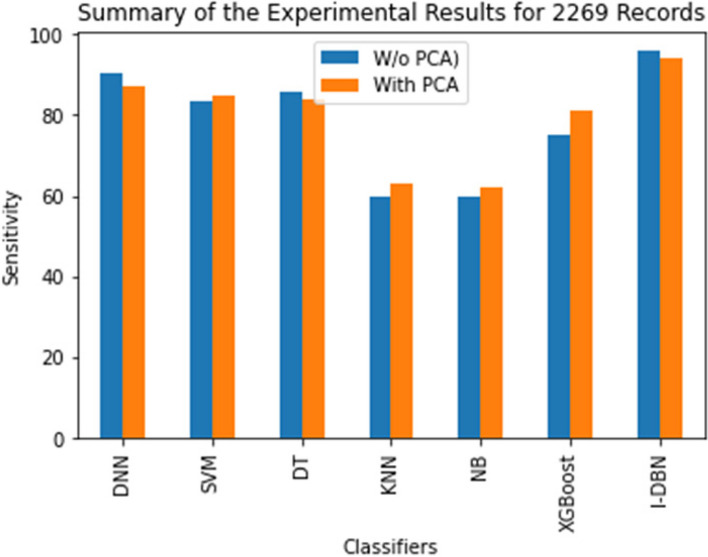
Performance Evaluation of I-DBN with other classifiers on sensitivity

**Fig. 9:**
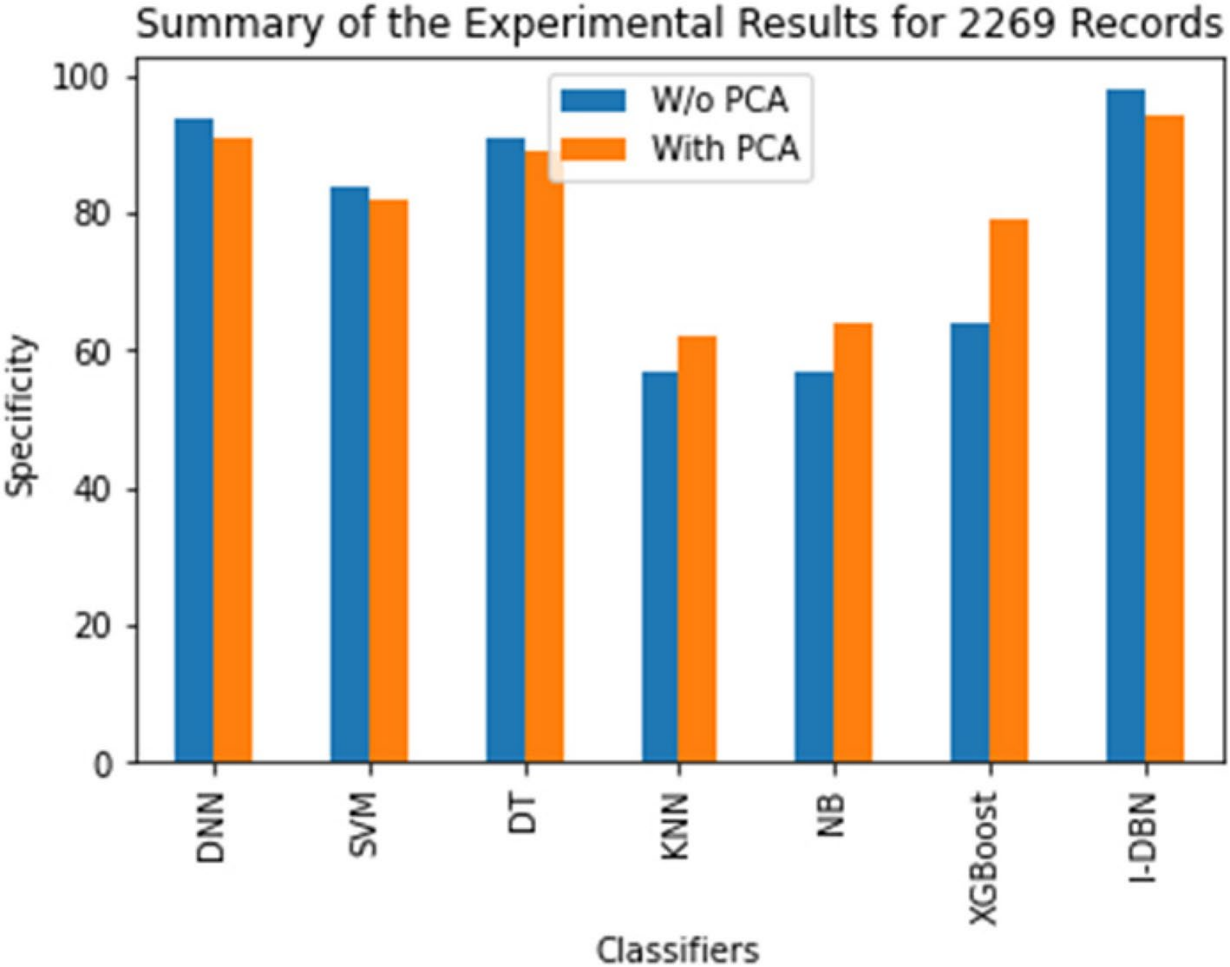
Performance Evaluation of DBN with other classifiers on specificity

The comparative performance of the I-DBN is assesses based on accuracy, precision, recall, specificity, and sensitivity metrics. The result of this proposed model is analyzed with the performance of conventional classifiers, Deep Neural Network (DNN), Support Vector Machines (SVM), Decision Tree (DT), K-Nearest Neighbour (KNN), Naive Bayes (NB), XGBoost are illustrated in Figs. 5, 6, 7, 8 and 9.

Figure 5 illustrates that the accuracy of the proposed system compared with various methods. The proposed I-DBN model achieved 98.95% of accuracy and 93.95% of accuracy has been achieved with DBN model. The classification accuracy of 98.95% is attained when applying proposed model which is 3.17% higher than DNN and 8.88% higher than the DNN with PCA.

Figure 6 depicts that the precision of the I-DBN compared with various methods. I-DBN achieves 97.61% of precision and 93.9% of precision has been achieved with DBN model. The precision of 97.61% is attained when applying I-DBN which is 3.05% higher than DNN and 7.61% higher than the DNN with PCA.

Figure 7 shows that the recall metric of the I-DBN compared with conventional methods. I-DBN attains 98.78% of recall and normal DBN attains 95.11% of recall. The recall of 98.78% is attained when applying I-DBN which is 4.18% higher than DNN and 7.78% higher than the DNN with PCA.

Similarly, Fig. 8 shows that the sensitivity metric of I-DBN compared with other models. It reaches 95.89%, 93.9% with I-DBN and DBN model respectively. The sensitivity of 95.89% is attained when applying I-DBN which is 5.59% higher than DNN and 8.89% higher than the DNN with PCA.

In Fig. 9 the specificity metric has been shown. I-DBN achieved 97.87% of specificity and DBN model achieves 94.14%. The specificity of 97.87% is attained when applying I-DBN which is 4.0% higher than DNN and 6.87% higher than the DNN with PCA. The observations say that the performance of I-DBN model is better after applying the feature selection technique with respect to the metrics accuracy, recall, precision, sensitivity, specificity.

This study introduces an approach by developing an I-DBN model for proactive CVD prevention. Unlike previous studies that largely focus on post-diagnosis CVD prediction, this research targets prevention of CVD before its onset, employing a method that innovatively combines feature selection, data augmentation, and classifier refinement. Traditional DBN applications often struggle with overfitting and rigidity in network design; however, this model overcomes these challenges by introducing randomized layer and hyperparameter selection, enhancing adaptability and optimizing network structure specifically for CVD prediction. Additionally, PSO is utilized for hyperparameter tuning, ensuring that DBN parameters are finely adjusted to achieve superior performance.

This work stands out with its application of PCA on an augmented dataset, which enhances DBN effectiveness by reducing feature dimensions while preserving model accuracy. This approach achieves a significant performance boost, reaching an accuracy of 98.95%, which surpasses prior models typically applied to original datasets without augmentation. Furthermore, unlike conventional classifiers such as SVM, DT, and XGBoost, the proposed I-DBN model not only demonstrates higher accuracy, precision, and recall but also maintains high specificity and sensitivity even with feature reduction.

The combined framework of I-DBN with PCA-augmented data and PSO optimization presents a comprehensive solution that outperforms state-of-the-art classifiers across multiple key performance metrics. This study thus represents a significant advancement over existing tools for CVD diagnosis and prevention.

The proposed I-DBN model incorporates several optimizations that reduce time complexity. PCA is applied for dimensionality reduction on the augmented dataset, thereby reducing the feature space and computational requirements for training. Furthermore, PSO is employed selectively for hyperparameter tuning to streamline the optimization process. By using CD for gradient estimation, the model further reduces training time by minimizing the calculations typically required for standard gradient descent. These optimizations allow the I-DBN model to maintain high predictive accuracy with manageable time complexity, supporting its suitability for real-time CVD prediction.

## Conclusion and scope of future work

In this study a combination of DBN-PCA-PSO named I-DBN model is proposed to classify the DR dataset. The dataset is taken from UCI ML database, a publicly available database [46, 57–59]. I-DBN used MinMaxScaler standardization method for removing the redundant data present in the raw dataset. It also removes the outliers and normalizes the data. In the next step PCA method reduces the dimensions of the features. Further, PSO algorithm is employed to perform parameter optimization for extracting suitable features from the dataset. These optimized features are given into I-DBN model which classifies the data with improved accuracy. To improvise the proposed model’s performance, the records were duplicated. 80:20 rules has been implemented to split the dataset for training phase and testing phase respectively. The result of I-DBN is estimated with respect to various standard performance metrics. Further, the performance of the proposed model is analysed with the principal machine learning approaches such as DNN, SVM, DT, KNN, NB and XGBoost. The results observed depict that the I-DBN model outperformed the above-mentioned algorithms. It is observed that the proposed model achieves accuracy of 98.95% than to the existing conventional models.

In the future work, the proposed model can be utilized for classifying different domains of datasets having high dimensional data. To further improvise the performance of the proposed model, it can be executed to huge volume of datasets with more number of attributes in the records of data. This approach can also be applied for DR image dataset in contributing towards better prediction/ classification of the disease in healthcare.

## Data Availability

The data presented in this study are openly available at 10.24432/C5XP4P.

## References

[1] Rajamhoana SP, Devi CA, Umamaheswari K, Kiruba R, Karunya K, Deepika R. (2018, July). Analysis of neural networks based heart disease prediction system. In *2018 11th International Conference on Human System Interaction (HSI)* (pp. 233–239). IEEE.

[2] Yeates K, Lohfeld L, Sleeth J, Morales F, Rajkotia Y, Ogedegbe O. A global perspective on cardiovascular disease in vulnerable populations. Can J Cardiol. 2015;31(9):1081–93. 26321432 10.1016/j.cjca.2015.06.035 PMC4787293

[3] Burger A, Pretorius R, Fourie CM, Schutte AE. The relationship between cardiovascular risk factors and knowledge of cardiovascular disease in African men in the North-West Province. Health sa Gesondheid. 2016;21:364–71. 10.1016/j.hsag.2016.07.003.

[4] Bergman HE, Reeve BB, Moser RP, Scholl S, Klein WM. Development of a comprehensive heart disease knowledge questionnaire. Am J Health Educ. 2011;42(2):74–87. 21720571 10.1080/19325037.2011.10599175 PMC3124098

[5] Gadekallu TR, Khare N, Bhattacharya S, Singh S, Maddikunta PKR, Srivastava G. (2020). Deep neural networks to predict diabetic retinopathy. J Ambient Intell Humaniz Comput, 1–14.

[6] Abràmoff MD, Garvin MK, Sonka M. Retinal imaging and image analysis. IEEE Rev Biomed Eng. 2010;3:169–208. 22275207 10.1109/RBME.2010.2084567 PMC3131209

[7] Sri RM, Rajesh V. (2015, December). Early detection of diabetic retinopathy from retinal fundus images using eigen value analysis. In 2015 International Conference on Control, Instrumentation, Communication and Computational Technologies (ICCICCT) (pp. 766–769). IEEE.

[8] Fong, D. S., Aiello, L., Gardner, T. W., King, G. L., Blankenship, G., Cavallerano, J. D., … Klein, R. (2004). Retinopathy in diabetes. Diabetes care, 27(suppl 1), s84–s87. 10.2337/diacare.27.2007.s84 14693935

[9] Kempen JH, O’Colmain BJ, Leske MC, Haffner SM, Klein R, Moss SE, et al. The prevalence of diabetic retinopathy among adults in the United States. Arch Ophthalmol (Chicago, Ill: 1960). 2004;122(4):552–63. 10.1001/archopht.122.4.552 15078674

[10] Duh EJ, Sun JK, Stitt AW. Diabetic retinopathy: current understanding, mechanisms, and treatment strategies. JCI insight. 2017;2(14):e93751. 28724805 10.1172/jci.insight.93751 PMC5518557

[11] Vinayakumar R, Alazab M, Soman KP, Poornachandran P, Al-Nemrat A, Venkatraman S. Deep learning approach for intelligent intrusion detection system. IEEE Access. 2019;7:41525–50.

[12] Yau JW, Rogers SL, Kawasaki R, Lamoureux EL, Kowalski JW, Bek T, Meta-Analysis for Eye Disease (META-EYE) Study Group. Global prevalence and major risk factors of diabetic retinopathy. Diabetes Care. 2012;35(3):556–64. 22301125 10.2337/dc11-1909 PMC3322721

[13] Schmidhuber J. Deep learning in neural networks: an overview. Neural Netw. 2015;61:85–117. 25462637 10.1016/j.neunet.2014.09.003

[14] Bengio Y, Courville A, Vincent P. Representation learning: a review and new perspectives. IEEE Trans Pattern Anal Mach Intell. 2013;35(8):1798–828. 23787338 10.1109/TPAMI.2013.50

[15] Smolensky P. Information processing in dynamical systems: foundations of harmony theory. Colorado Univ at Boulder Dept of Computer Science; 1986.

[16] Zhang YD, Wang S, Dong Z. Classification of Alzheimer disease based on structural magnetic resonance imaging by kernel support vector machine decision tree. Progress Electromagnet Res. 2014;144:171–84.

[17] Sahlsten J, Jaskari J, Kivinen J, Turunen L, Jaanio E, Hietala K, Kaski K. Deep learning fundus image analysis for diabetic retinopathy and macular edema grading. Sci Rep. 2019;9(1):1–11. 31341220 10.1038/s41598-019-47181-w PMC6656880

[18] Krause J, Gulshan V, Rahimy E, Karth P, Widner K, Corrado GS, et al. Grader variability and the importance of reference standards for evaluating machine learning models for diabetic retinopathy. Ophthalmology. 2018;125(8):1264–72. 29548646 10.1016/j.ophtha.2018.01.034

[19] Li X, Pang T, Xiong B, Liu W, Liang P, Wang T. (2017, October). Convolutional neural networks based transfer learning for diabetic retinopathy fundus image classification. In 2017 10th international congress on image and signal processing, biomedical engineering and informatics (CISP-BMEI) (pp. 1–11). IEEE.

[20] Lahmiri S, Shmuel A. Variational mode decomposition based approach for accurate classification of color fundus images with hemorrhages. Opt Laser Technol. 2017;96:243–8.

[21] Gulshan, V., Peng, L., Coram, M., Stumpe, M. C., Wu, D., Narayanaswamy, A., … Webster,D. R. (2016). Development and validation of a deep learning algorithm for detection of diabetic retinopathy in retinal fundus photographs. Jama, 316(22), 2402–2410. 10.1001/jama.2016.17216 27898976

[22] Castellano G, Castiello C, Mencar C, Vessio G. (2020, January). Crowd detection for drone safe landing through fully-convolutional neural networks. In International conference on current trends in theory and practice of informatics (pp. 301–312). Springer, Cham.

[23] Swapna G, Kp S, Vinayakumar R. Automated detection of diabetes using CNN and CNN-LSTM network and heart rate signals. Procedia Comput Sci. 2018;132:1253–62.

[24] Swapna G, Vinayakumar R, Soman KP. Diabetes detection using deep learning algorithms. ICT Express. 2018;4(4):243–6.

[25] Swapna G, Soman KP, Vinayakumar R. Diabetes detection using ecg signals: an overview. Deep Learning Techniques for Biomedical and Health Informatics; 2020. pp. 299–327.

[26] Poplin R, Varadarajan AV, Blumer K, Liu Y, McConnell MV, Corrado GS, Webster DR. Prediction of cardiovascular risk factors from retinal fundus photographs via deep learning. Nat Biomedical Eng. 2018;2(3):158–64. 10.1038/s41551-018-0195-0 31015713

[27] Oh K, Kang HM, Leem D, Lee H, Seo KY, Yoon S. Early detection of diabetic retinopathy based on deep learning and ultra-wide-field fundus images. Sci Rep. 2021;11(1):1–9. 33479406 10.1038/s41598-021-81539-3 PMC7820327

[28] Arcadu F, Benmansour F, Maunz A, Willis J, Haskova Z, Prunotto M. Deep learning algorithm predicts diabetic retinopathy progression in individual patients. NPJ Digit Med. 2019;2(1):1–9. 31552296 10.1038/s41746-019-0172-3 PMC6754451

[29] Salakhutdinov R. (2009). Learning deep generative models [Ph. D. thesis]. University of Toronto.

[30] Ali SA, Raza B, Malik AK, Shahid AR, Faheem M, Alquhayz H, Kumar YJ. An optimally configured and Improved Deep Belief Network (OCI-DBN) Approach for Heart Disease Prediction based on ruzzo–tompa and stacked genetic algorithm. IEEE Access. 2020;8:65947–58.

[31] Wang X, Wang W, Ren H, Li X, Wen Y. Prediction and analysis of risk factors for diabetic retinopathy based on machine learning and interpretable models. Heliyon. 2024;10(109). 10.1016/j.heliyon.2024.e29497. 10.1016/j.heliyon.2024.e29497 PMC11064081 38699007

[32] Smith A, Gupta R, Liu Y. Deep learning for diabetic retinopathy progression and cardiovascular risk assessment from retinal images. BMJ Open Ophthalmol. 2023;8(2):34–47.

[33] Jones D, Patel S. Retinal biomarkers for systemic health: a machine learning perspective on diabetic retinopathy and cardiovascular disease. J Biomed Inform. 2023;133:104235.

[34] Garcia M, Huang J. Machine learning-based cardiovascular risk assessment in diabetic populations using diabetic retinopathy data. Diabetes Vasc Dis Res. 2024;21(1):56–70.

[35] Deepa R, Sivasamy A. Advancements in early detection of diabetes and diabetic retinopathy screening using artificial intelligence. AIP Adv. 2023;13(115307): 115307. 10.1063/5.0172226.

[36] Deepa R, Arunkumar S, Jayaraj V, Sivasamy A. Healthcare’s new frontier: AI-driven early cancer detection for improved well-being. AIP Adv. 2023;13(115331): 115331. 10.1063/5.0177640.

[37] Elkhenini H, Wong TY, Buitendyk M. Diabetic retinopathy as a predictive biomarker for cardiovascular events in type 2 diabetes. Diabetes Care. 2024;47(2):311–8.

[38] Chen L, Zhang X, Zhou H. Cardiovascular health assessment using retinal images and AI. J Biomed Inform. 2023;145:103837.

[39] Happ C, Greven S. Multivariate functional principal component analysis for data observed on different (dimensional) domains. J Am Stat Assoc. 2018;113(522):649–59.

[40] Shahzad F, Masood S, Khan NK. Probabilistic opposition-based particle swarm optimization with velocity clamping. Knowl Inf Syst. 2014;39(3):703–37.

[41] Yu L, Wang S, Lai KK, Wen F. A multiscale neural network learning paradigm for financial crisis forecasting. Neurocomputing. 2010;73(4–6):716–25.

[42] Wang XH, He YG, Li TZ. Neural network algorithm for designing FIR filters utilizing frequency-response masking technique. J Comput Sci Technol. 2009;24(3):463–71.

[43] Gadekallu TR, Khare N, Bhattacharya S, Singh S, Reddy Maddikunta PK, Ra IH, Alazab M. Early detection of diabetic retinopathy using PCA-firefly based deep learning model. Electronics. 2020;9(2):274.

[44] Hemanth DJ, Deperlioglu O, Kose U. An enhanced diabetic retinopathy detection and classification approach using deep convolutional neural network. Neural Comput Appl. 2020;32(3):707–21.

[45] Fischer A, Igel C. Training restricted Boltzmann machines: an introduction. Pattern Recogn. 2014;47(1):25–39.

[46] Soedamah-Muthu SS, Chaturvedi N, Witte DR, Stevens LK, Porta M, Fuller JH. Relationship between risk factors and mortality in type 1 diabetic patients in Europe: the EURODIAB prospective complications study (PCS). Diabetes Care. 2008;31(7):1360–6. 18375412 10.2337/dc08-0107 PMC2453640

[47] Dinneen SF, Gerstein HC. The association of microalbuminuria and mortality in non—insulin-dependent diabetes mellitus: a systematic overview of the literature. Arch Intern Med. 1997;157(13):1413–8. 9224218

[48] Sudha V, Karthikeyan C. Analysis of diabetic retinopathy using naive bayes classifier technique. Int J Eng Technol. 2018;7(221):440–2.

[49] Jolliffe IT. Principal component analysis. Technometrics. 2003;45(3):276.

[50] Song F, Guo Z, Mei D. (2010, November). Feature selection using principal component analysis. In 2010 international conference on system science, engineering design and manufacturing informatization (Vol. 1, pp. 27–30). IEEE.

[51] Jain D, Singh V. An efficient hybrid feature selection model for dimensionality reduction. Procedia Comput Sci. 2018;132:333–41.

[52] Hinton GE, Osindero S, Teh YW. A fast learning algorithm for deep belief nets. Neural Comput. 2006;18(7):1527–54. 16764513 10.1162/neco.2006.18.7.1527

[53] Mohamed A, Dahl GE, Hinton GE. Deep belief networks for phone recognition,[in:] NIPS Workshop on Deep Learning for Speech Recognition and related applications. Whistler, BC, Canada; 2009.

[54] Xiaohui, Hu. (2006) Particle Swarm Optimization, www.swarmintelligence.org .

[55] Clerc M, Kennedy J. The particle swarm-explosion, stability, and convergence in a multidimensional complex space. IEEE Trans Evol Comput. 2002;6(1):58–73.

[56] Wang D, Tan D, Liu L. Particle swarm optimization algorithm: an overview. Soft Comput. 2018;22(2):387–408.

[57] Klein R, Klein BE, Moss SE, Cruickshanks KJ. Association of ocular disease and mortality in a diabetic population. Arch Ophthalmol. 1999;117(11):1487–95. 10565517 10.1001/archopht.117.11.1487

[58] Van Hecke MV, Dekker JM, Stehouwer CD, Polak BC, Fuller JH, Sjolie AK, Chaturvedi N. Diabetic retinopathy is associated with mortality and cardiovascular disease incidence: the EURODIAB prospective complications study. Diabetes Care. 2005;28(6):1383–9. 15920056 10.2337/diacare.28.6.1383

[59] Miettinen H, Haffner SM, Lehto S, Rönnemaa T, Pyörälà K, Laakso M. Retinopathy predicts coronary heart disease events in NIDDM patients. Diabetes Care. 1996;19(12):1445–8. 8941482 10.2337/diacare.19.12.1445

